# Deep Learning Algorithm Trained on Brain Magnetic Resonance Images and Clinical Data to Predict Motor Outcomes of Patients With Corona Radiata Infarct

**DOI:** 10.3389/fnins.2021.795553

**Published:** 2022-01-03

**Authors:** Jeoung Kun Kim, Min Cheol Chang, Donghwi Park

**Affiliations:** ^1^Department of Business Administration, School of Business, Yeungnam University, Gyeongsan, South Korea; ^2^Department of Rehabilitation Medicine, College of Medicine, Yeungnam University, Gyeongsan, South Korea; ^3^Department of Physical Medicine and Rehabilitation, Ulsan University Hospital, University of Ulsan College of Medicine, Ulsan, South Korea

**Keywords:** artificial intelligence, deep learning, corona radiate, cerebral infarction, motor outcome

## Abstract

The early and accurate prediction of the extent of long-term motor recovery is important for establishing specific rehabilitation strategies for stroke patients. Using clinical parameters and brain magnetic resonance images as inputs, we developed a deep learning algorithm to increase the prediction accuracy of long-term motor outcomes in patients with corona radiata (CR) infarct. Using brain magnetic resonance images and clinical data obtained soon after CR infarct, we developed an integrated algorithm to predict hand function and ambulatory outcomes of the patient 6 months after onset. To develop and evaluate the algorithm, we retrospectively recruited 221 patients with CR infarct. The area under the curve of the validation set of the integrated modified Brunnstrom classification prediction model was 0.891 with 95% confidence interval (0.814–0.967) and that of the integrated functional ambulatory category prediction model was 0.919, with 95% confidence interval (0.842–0.995). We demonstrated that an integrated algorithm trained using patients’ clinical data and brain magnetic resonance images obtained soon after CR infarct can promote the accurate prediction of long-term hand function and ambulatory outcomes. Future efforts will be devoted to finding more appropriate input variables to further increase the accuracy of deep learning models in clinical applications.

## Introduction

Despite the progress in drug development and disease management, the mortality and disability rates of ischemic strokes remain high (Donkor, 2018; Kuriakose and Xiao, 2020). In fact, the overall disability rate has been reported as high as 75% in ischemic stroke survivors (Ovbiagele and Nguyen-Huynh, 2011; Donkor, 2018). Among the various disabilities after the onset of ischemic stroke, motor deficiency in patients is one of the most critical sequelae (Palumbo et al., 1978; Alawieh et al., 2018).

Among the various ischemic stroke lesions, those affecting the corona radiata (CR) and posterior limb of the internal capsule are often associated with poor motor outcomes (Turhan et al., 2006; Frenkel-Toledo et al., 2019). For example, Shelton and Reding (2001) reported that only 5.9% of patients with a CR infarct showed isolated movement recovery of the upper limb, which was much lower than the 75% recovery of patients with cortical infarct. Therefore, patients who suffer from CR infarct require an accurate prediction of the extent of long-term motor recovery at an early stage to ensure that clinicians can establish individual rehabilitation strategies that are conducive to improved motor outcomes.

Deep learning is a recent artificial intelligence technique in which a system learns patterns and rules from the available data (Lee et al., 2020; Choo et al., 2021; Sarker, 2021). Thus, it has been increasingly applied in the clinical field. In particular, deep learning can be applied to accurately predict the long-term motor outcomes of stroke patients (Kamal et al., 2018; Heo et al., 2019).

To develop algorithms including those for deep learning, most existing studies have focused on either clinical or imaging data (Choo et al., 2021; Kim et al., 2021a,b). However, developing algorithms using clinical data requires a large number of variables, and each hospital collects different types of clinical data. Consequently, an algorithm developed using specific clinical data is difficult to utilize in general clinical practice. Regarding imaging data, as most hospitals use imaging modalities such as brain magnetic resonance (MR) imaging for diagnosing ischemic stroke, high data consistency and algorithm applicability can be achieved (Davis et al., 2006). Nevertheless, deep learning based only on brain MR images tends to show low prediction accuracy (Kim et al., 2021b).

In this study, we used both clinical parameters and brain MR images as inputs to develop a deep learning algorithm that can increase the prediction accuracy of long-term motor abilities of patients with CR infarct.

## Materials and Methods

### Patients

A total of 221 consecutive patients (mean age, 65.0 ± 11.9 years; 115 males, 106 females; time to transfer or admission, 30.2 ± 72.2 days; initial modified Brunnstrom classification—MBC within 7–30 days of infarct onset, 2.4 ± 1.8; initial functional ambulation category—FAC within 7–30 days of infarct onset, 1.0 ± 1.2) who suffered CR infarct and underwent stroke rehabilitation in a single university hospital from January 2003 to January 2020 were retrospectively recruited in this study (Table 1). The inclusion criteria were as follows: (1) a patient’s first ever stroke; (2) age > 20 years; (3) hemiparesis or hemiplegia hemiparesis after cerebral infarction in their CR; (4) brain MR imaging conducted within 30 days after stroke onset; (4) MBC ≤ 4 and FAC ≤ 3 within 7–30 days of infarct onset; (5) motor function checked 6 months after stroke onset; and (6) absence of other severe medical conditions (e.g., cardiac problems and pneumonia) between the onset and final evaluation.

**TABLE 1:** Patient demographic and clinical data collected within 7–30 days of infarct onset.

**Demographic data**	
Number of patients, n	221
Age, years	65.0 ± 11.9
**Clinical data within 7–30 days of infarct onset**	
MBC	2.4 ± 1.8
FAC	1.0 ± 1.2
MRC of shoulder abductor	1.5 ± 1.4
MRC of elbow flexor	1.6 ± 1.5
MRC of finger flexor	1.3 ± 1.5
MRC of finger extensor	1.1 ± 1.5
MRC of hip flexor	2.0 ± 1.4
MRC of knee extensor	2.1 ± 1.5
MRC of ankle dorsiflexor	1.5 ± 1.5

*MBC, modified Brunnstrom classification; FAC, functional ambulation category; MRC, medical research council.*

The study protocol was approved by the Institutional Research Board of Yeungnam University Hospital (No. 2019-10-008). The requirement for informed consent was waived because we used de-identified retrospective data, as confirmed by our institutional review board. This study was conducted in accordance with the principles of the Declaration of Helsinki.

### Images for the Deep Learning Algorithm

Three T2-axial consecutive brain MR images obtained from every cerebral infarction patient were investigated in our study. The images were taken at the lateral ventricle level of the body so that CR fibers passing above the internal capsule can be observed. The MR images obtained on the day closest to the date of transfer to the physical medicine and rehabilitation department within 30 days after cerebral infarct onset were used for the development of the algorithm.

### Clinical Data for the Deep Learning Algorithm

We investigated 11 input variables at the early stage after the onset of CR infarction: age, sex, MBC, FAC, and the Medical Research Council’s score for muscle power of the shoulder abductor, elbow flexor, finger flexor, finger extensor, hip flexor, knee extensor, and ankle dorsiflexor on the affected side. For convenient use in clinical practice, data that could be easily assessed by clinicians were selected.

### Motor Outcome at 6 Months

The MBC score was obtained as the motor outcome of the affected hand, and the FAC score, which quantifies ambulation, was obtained from the affected leg.

The MBC quantifies the motor function of the affected upper limb from 1 to 6, with higher scores indicating better hand function (Huang et al., 2016). The MBC scores of the upper limbs were used in the analysis. We separated the MBC score 6 months after infarction onset as follows: favorable outcomes for lower limbs given an MBC score of at least 5, indicating minimal spasticity with slightly increased tone, and those given an MBC score below 5, indicating the ability to initiate non-synergistic movement. Among the 221 patients, one patient’s MBC was not assessed because of an ulnar fracture. Therefore, data from 220 patients were used to train and evaluate the algorithm to predict upper limb motor function.

The FAC score is evaluated based on the degree of assistance required during a 15 m walk (Mehrholz et al., 2007). The FAC is categorized as follows: (0) Non-ambulatory, (1) continuous support from one person necessary, (2) intermittent support from one person necessary, (3) requirement of verbal supervision only, (4) assistance required on stairs and uneven surfaces, and (5) ability to walk independently anywhere. We evaluated the FAC score 6 months after infarction onset as follows: favorable outcomes for lower limbs given by an FAC score of at least 4, indicating the ability to walk without a keeper’s assistance, and a score below 4, indicating poor outcomes for the lower limbs (Smith et al., 2017; Stinear et al., 2019).

### Deep Learning Algorithms

For brain MR image analysis, we used a convolutional neural network (CNN) implemented using the Python programming language. TensorFlow 2.4, Keras, and scikit-learn 0.23.2 libraries were used to train the deep learning model. EfficientNet CNN models were used for both MBC and FAC prediction.

**FIGURE 1:**
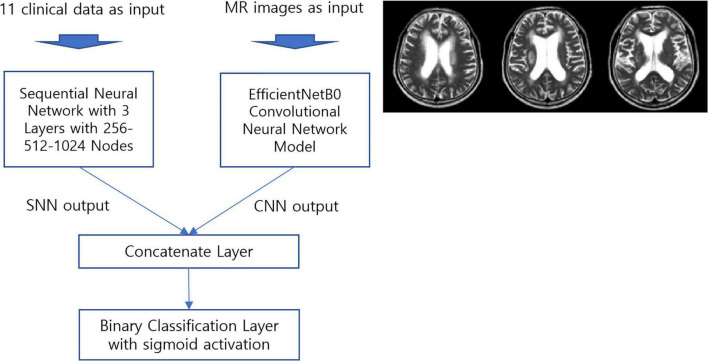
Proposed prediction model based on deep learning. (MR, magnetic resonance; SNN, sequential neural network; CNN, convolutional neural network).

**FIGURE 2:**
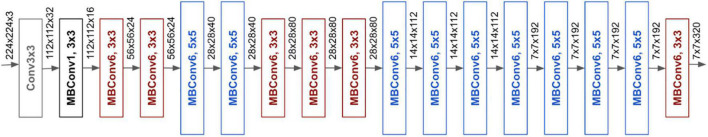
Baseline architecture of EfficientNetB0 (Tan and Le, 2019a).

For clinical data analysis, we used a sequential neural network with three layers containing 256, 512, and 1,024 neurons. The details of the model and its performance are provided in Table 2. The integrated prediction model was composed of the EfficientNetB0 (Tan and Le, 2019b) CNN model and a sequential neural network. A diagram of the resulting and baseline architectures of EfficientnetB0 are presented in Figures 1, 2. EfficientNet models are known to achieve improved accuracy while reducing the number of parameters compared to other CNNs (Tan and Le, 2019a). EfficientNetB0 has the advantage of fast learning speed and reduced overfitting in MR image training because it has fewer parameters than other CNN models.

**TABLE 2:** Performance of deep learning algorithm for predicting motor outcome after corona radiata infarct.

	MBC prediction model	FAC prediction model
Sample size (patients)	154 For training (462 images, 70%), 66 for validation (198 images, 30%) Sample ratio: Poor 52.7% (0), good 47.3% (1)	154 For training (462 images, 70%), 67 for validation (201 images, 30%) Sample ratio: Poor 54.3% (0), good 45.7% (1)
CNN model	Model for MR images	
	- EfficientNetB0 with fine-tuning - SGD optimizer, ReLU activation - Data augmentation and dropout for regularization - Image of size 256 × 256	- EfficientNetB0 with fine-tuning - RMSProp optimizer, ReLU activation - Data augmentation and dropout for regularization - Image of size 256 × 256
Sequential neural network model	Model for clinical data - 3 hidden layers with 256-512-1,024 neurons - SGD optimizer, ReLU activation - Batch normalization for regularization - 11 clinical variables as inputs	- 3 hidden layers with 256-512-1,024 neurons - RMSProp optimizer, ReLU activation - Batch normalization for regularization - 11 clinical variables as inputs
Integrated prediction model	Concatenated model with CNN and sequential neural network outputs - MBC and FAC prediction with three images and clinical data per patient - Binary classification with sigmoid activation	
Decision criteria for integrated prediction model	Poor (0): less than 3 “good” predictions; good (1): 3 “good” predictions	
Integrated prediction model performance	MBC prediction accuracy of 90.91% on training data Training AUC of 0.907 with 95% CI [0.861–0.953] MBC prediction accuracy of 89.39% on validation data Validation AUC of 0.891 with 95% CI [0.814–0.967]	FAC prediction accuracy of 91.6% on training data Training AUC of 0.935 with 95% CI [0.896–0.975] FAC prediction accuracy of 91.1% on validation data Validation AUC of 0.919 with 95% CI [0.842–0.995]

*MBC, modified Brunnstrom classification; FAC, functional ambulation category; MR, magnetic resonance; CNN, convolutional neural network; SNN, sequential neural network; SGD, stochastic gradient descent; ReLU, rectified linear unit; RMSProp, root mean square propagation; AUC, area under the curve; CI, confidence interval.*

To compare the performance of our integrated prediction model, we used the ResNet50 CNN model (He et al., 2016) for MR images and a random forest model for clinical data. ResNet is a well-known CNN model as the winner of the 2015 ILSVRC (ImageNet Large Scale Visual Recognition Challenge). Random forest is a classification algorithm frequently used in machine learning. We compared the performance of ResNet50, random forest, and our integrated prediction model, which uses both MR images and clinical data as inputs. The details are presented in Table 3.

**TABLE 3:** Performance comparison among three models for predicting motor outcome after corona radiata infarct.

	MBC prediction model	FAC prediction model
Integrated model	Integrated model with both MR images and clinical data as input EfficientNetB0 for MR images Sequential neural network with three layers (1024-512-256) for clinical data Accuracy: 90.91% on training data, 89.39% on validation data AUC on validation data: 0.891 with 95% CI [0.814–0.967]	Integrated model with both MR images and clinical data as input EfficientNetB0 for MR images Sequential neural network with three layers (1024-512-256) for clinical data Accuracy: 91.6% on training data, 91.1% on validation data AUC on validation data: 0.919 with 95% CI [0.842–0.995]
CNN model for MR images	ResNet50 CNN model with MR images as input Training parameters: SGD optimizer, ReLU activation, learning rate 6e-05, batch size 4, fine tuning with last 10 layers trained, binary classification with sigmoid activation Accuracy: 87.8% on training data, 65.66% on validation data AUC on validation data: 0.636 with 95% CI [0.568–0.704]	ResNet50 CNN model for MR images Training parameters: SGD optimizer, ReLU activation, learning rate 8e-05, batch size 8, full layers trained, binary classification with sigmoid activation Accuracy: 98.05% on training data, 80.1% on validation data AUC on validation data: 0.638 with 95% CI [0.517–0.626]
ML model for clinical data	Random forest model with clinical data as input Scikit learn GridSearchCV for hyper parameter optimization Training parameters: Max_depth 4, min_sample_leaf 4, min_smaples_split 12, n_estimators 100, random_state 99 Mean accuracy: 83.66% on training data, 74.24% on validation data AUC on validation data: 0.754 with 95% CI [0.694–0.814]	Random forest model with clinical data as input Scikit learn GridSearchCV for hyper parameter optimization Training parameters: Max_depth 4, min_sample_leaf 14, min_smaples_split 4, n_estimators 10, random_state 99 Mean accuracy: 85.06% on training data, 80.6% on validation data AUC on validation data: 0.638 with 95% CI [0.567–0.710]

*ML, machine learning; MBC, modified Brunnstrom classification; FAC, functional ambulation category; MR, magnetic resonance; CNN, convolutional neural network; SGD, stochastic gradient descent; ReLU, rectified linear unit; RMSprop, root mean square propagation; AUC, area under the curve; CI, confidence interval.*

To predict the motor outcome of each patient, the integrated prediction model used three brain MR images and the patient’s clinical data as inputs and returned three predictions, one per MR image combined with the clinical data. If the three predictions were accurate, the final judgment was considered suitable, and if any prediction was inaccurate, it was considered poor.

We conducted an ablation study of our model to understand critical components. Two core parts of the proposed integrated model, the image model (CNN) and the clinical data model (SNN), were separately trained using the same parameters as the integrated model, and their performances were compared. Table 4 shows our ablation study results in detail.

**TABLE 4:** Ablation study of the integrated prediction model.

	MBC prediction model	FAC prediction model
Integrated model	Integrated model with both MR images and clinical data as input EfficientNetB0 for MR images Sequential neural network with three layers (1024-512-256) for clinical data Accuracy: 90.91% on training data, 89.39% on validation data AUC: 0.907 on training data, 0.891 on validation data	Integrated model with both MR images and clinical data as input EfficientNetB0 for MR images Sequential neural network with three layers (1024-512-256) for clinical data Accuracy: 91.6% on training data, 91.1% on validation data AUC: 0.935 on training data, 0.919 on validation data
CNN model only	EfficientNetB0 CNN model with fine tuning MR images as input Training parameters: SGD optimizer, ReLU activation, lr 8e-06, dr 0.2, bs 64, binary classification with sigmoid activation Accuracy: 73.8% on training data, 63.6% on validation data AUC: 0.974 on training data, 0.619 on validation data	EfficientNetB0 CNN model with fine tuning MR images as input Training parameters: RMSProp optimizer, ReLU activation, lr 8e-06, dr 0.25, bs 64, binary classification with sigmoid activation Accuracy: 72.2% on training data, 63.0% on validation data AUC: 0.852 on training data, 0.662 on validation data:
SNN model only	SNN with clinical data 3 Hidden layers with 256-512-1024 neurons Training parameters: SGD optimizer, ReLU activation, lr 8e-06, dr 0.2, bs 64, binary classification with sigmoid activation Batch normalization and dropout for regularization 11 Clinical variables as inputs Accuracy: 94.2% on training data, 83.3% on validation data AUC: 0.980 on training data, 0.845 on validation data	SNN with clinical data 3 Hidden layers with 256-512-1024 neurons Training parameters: RMSProp optimizer, ReLU activation, lr 8e-06, dr 0.25, bs 64, binary classification with sigmoid activation Batch normalization and dropout for regularization 11 Clinical variables as inputs Accuracy: 96.3% on training data, 80.6% on validation data AUC: 0.992 on training data, 0.785 on validation data:

*ML, machine learning; MBC, modified Brunnstrom classification; FAC, functional ambulation category; MR, magnetic resonance; CNN, convolutional neural network; SNN, sequential neural network; SGD, stochastic gradient descent; ReLU, rectified linear unit; RMSProp, root mean square propagation; AUC, area under the curve; CI, confidence interval; lr, learning rate; dr, dropout rate; bs, batch size.*

### Statistical Analysis

The analysis for the receiver operating characteristic curve was performed, and the area under the curve (AUC) was calculated using the scikit-learn library. The confidence interval (CI) for the AUC was calculated using the method developed by DeLong et al. (1988).

## Results

**FIGURE 3:**
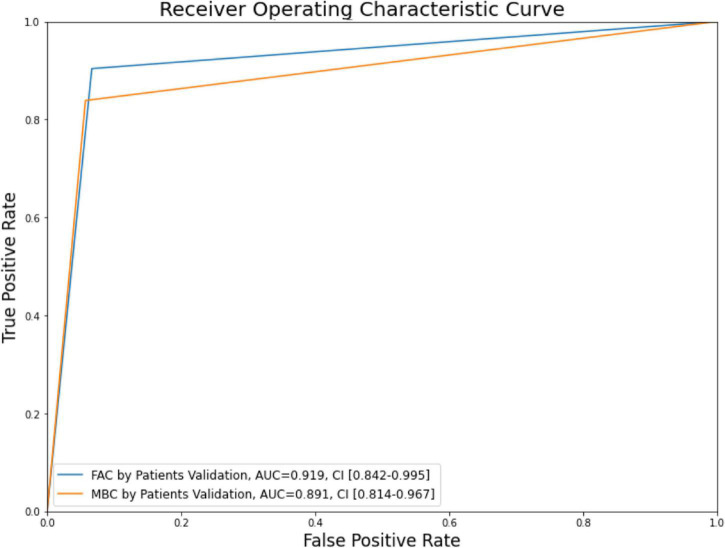
Modified Brunnstrom classification (MBC) and functional ambulation category (FAC) receiver operating characteristic curve and area under the curve (AUC) on the validation set.

From the 221 patients included in this study, we obtained AUC values for the validation set for the integrated MBC and FAC prediction models of 0.891 with 95% CI (0.814–0.967) and 0.919 with 95% CI (0.842–0.995), respectively (Table 2 and Figure 3).

Ablation study shows that the clinical data model outperformed the image model. The clinical data model’s prediction accuracy and AUC on validation data are 83.3% and 0.845 for MBC, 80.6%, and 0.785 for FAC. On the other hand, the image model performance on validation data showed an accuracy of 63.6% and AUC 0.619 for MBC, accuracy 63%, and AUC 0.662 for FAC.

## Discussion

We evaluated the effectiveness of a deep learning algorithm for predicting motor outcomes of patients 6 months after CR infarct onset. We developed integrated MBC and FAC models by combining a CNN to process brain MR images and a sequential neutral network to process 11 variables of the clinical data. To the best of our knowledge, this is the first study conducted on deep learning applied to the prediction of motor outcomes using brain MR images and clinical data. Unlike previous studies, we separately predicted the long-term outcomes of upper and lower limb motor outcomes by developing models for MBC and FAC prediction.

For the prediction of upper limb function 6 months after CR infarct onset, the AUC of the integrated MBC prediction model was 0.891 for the validation set. For the prediction of lower limb function (ambulatory function) 6 months after onset, the AUC of the integrated FAC prediction model was 0.919 for the validation set. Considering that AUC values of 0.7–0.8 are acceptable, 0.8–0.9 are excellent, and those above 0.9 are outstanding, the ability of the proposed prediction models for the prognosis of upper limb function is excellent to outstanding.

To date, for the early prediction of long-term motor outcomes in stroke patients, clinicians have used several tools, such as prognostic scoring systems (e.g., ASTRAL and ICHOP scores), using conventional brain MR images and clinical data (Papavasileiou et al., 2013; Gupta et al., 2017). Although the prediction accuracy of motor outcomes in prognostic scoring systems varies, most of these tools use a limited set of parameters to calculate the scores. Moreover, in clinical practice, a specific prognostic scoring system globally may be difficult to apply because different clinical data are frequently collected depending on the medical center.

Artificial intelligence has recently been applied in the prediction of motor outcomes after stroke onset. Heo et al. (2019) predicted the modified Rankin score 3 months after ischemic stroke using a deep neural network trained on data from 2604 acute ischemic stroke patients. They used 38 clinical parameters as inputs, including patient demographics, stroke subtypes, initial scores in the National Institutes of Health Stroke Scale, and time from onset to admission. The resulting AUC for predicting the motor outcome was 0.888. Sale et al. (2018) investigated the predictability of improvement in motor function due to rehabilitation early after stroke onset. The authors used data from 55 patients collected at the time of admission to the Department of Rehabilitation Medicine and discharge. They then predicted the Barthel index and functional independence measure using a linear support vector machine. The predictions and measurements showed a high correlation of 0.75–0.81. Similarly, Lin et al. (2018) constructed a prognostic model for functional outcomes using a support vector machine from the clinical data of 313 stroke patients. They used various functional measurement outcomes early after stroke as inputs, such as the modified Rankin score and Barthel index, gait speed, and results of the Mini-Mental State Exam, and then they predicted the Barthel index at discharge. The AUC for predicting the motor outcome was 0.774. Kim et al. (2021b) used brain MR imaging data obtained early after infarction to develop a CNN to predict the ambulatory outcome 6 months after onset in patients with CR infarction. They classified the outcome of the ambulatory function into two categories: (1) Favorable outcome of ambulatory function (FAC score of at least 4 with ability to walk without a guardian’s assistance) and (2) poor outcome of ambulatory function (FAC score < 4). Using a CNN, they reported an AUC of 0.751 with 95% CI (0.649–0.852) to predict ambulatory function.

Most existing methods predict broad functional scores, such as the modified Rankin score, functional independence measure, or Barthel index, with the exception of Kim et al. (2021b), who studied ambulation functions using the FAC. However, it is necessary to evaluate the functional prognosis of both the upper and lower limbs separately to plan early an effective rehabilitation strategy. Unlike previous studies, we separately predicted the functions of the upper and lower limbs using integrated MBC and FAC models, respectively. The AUC obtained by Heo et al. (2019) tended to be higher (AUC = 0.888) than that reported in other studies. However, they used only six clinical data sources (i.e., age, scores on the National Institutes of Health Stroke Scale, onset to admission delay, visual field defect, glucose, and level of consciousness) as inputs for predicting motor prognosis, thus neglecting image data. Although their AUC was similar to that obtained in our study, they did not assess the specific hand or ambulatory function, but only provided a general function score on the modified Rankin scale. If the hand and ambulatory motor outcomes were separately predicted as in our study, a lower prediction accuracy would be expected because only six variables were used by Heo et al. (2019). In contrast, Lin et al. (2018) used several inputs obtained early after stroke, including the modified Rankin score, Barthel index, functional oral intake score, mini nutritional assessment results, EQ-5D-5L (the European quality of life) index, instrumental activities of daily living scale score, Berg balance test score, gait speed, 6-min walk test score, Fugl–Meyer upper limb assessment score, modified Fugl–Meyer sensory assessment score, results of the Mini-Mental State Exam, usage of motor activity log, and results of the Concise Chinese Aphasia Test. However, their models are not suitable for clinical practice because it is time-consuming and cumbersome to obtain all the input values. Moreover, their AUC (0.774) was lower than that obtained in our study.

In this study, by using brain MR images and clinical data that can be easily assessed and obtained by clinicians, such as the Medical Research Council’s score of the affected limb, we developed deep learning algorithms with high prediction accuracy and applicability. Kim et al. (2021b) used only brainMR images, which may be advantageous for clinical applicability because most hospitals use such images in the diagnosis of cerebral infarction. Nevertheless, the AUC of 0.751 obtained by Kim et al. (2021b) revealed an unsatisfactory prediction accuracy. In contrast, we developed integrated deep learning algorithms using both brain MR images and clinical data, which are easily obtained in clinical practice, and achieved a high prediction accuracy for the long-term motor outcomes of hand and ambulatory functions. Because clinical data (i.e., age, sex, MBC, FAC, and MRC) used as inputs are simple and commonly measured in clinical practice and brain MRI is a vital tool for diagnosing stroke, we believe that our algorithm can be easily applied in other hospitals.

Our study was limited in that we used a small sample size of patient data to train the deep learning model. More samples from stroke patients are likely to increase the prediction accuracy of the model. Additionally, only patients with CR infarcts were included in this study. Including the data from patients with various other brain lesions can improve the generalization ability of the proposed deep learning algorithm for predicting long-term motor outcomes after stroke. Finally, we did not include factors affecting motor prognosis after stroke, such as stroke treatment at the acute stage and the duration of rehabilitation, as input variables in the algorithm. In future work, more appropriate input variables to further increase the prediction accuracy of deep learning algorithms should be selected for application in clinical practice.

## Conclusion

We demonstrated that integrated deep learning algorithms trained using patients’ clinical data and brain MR images obtained early after CR infarction can contribute to accurately predicting long-term hand function and ambulatory outcomes. The algorithm achieved a suitably high accuracy and thus can support clinicians in the prediction of long-term hand function and ambulatory outcomes.

## Data Availability Statement

The original contributions presented in the study are included in the article/supplementary material, further inquiries can be directed to the corresponding author/s.

## Ethics Statement

The Study Protocol was approved by the Institutional Review Board of Yeungnam University Hospital (protocol number: YUMC 2019-10-008, approval Date: Oct 31, 2019). Written informed consent for participation was not required for this study in accordance with the national legislation and the institutional requirements.

## Author Contributions

All authors listed have made a substantial, direct, and intellectual contribution to the work, and approved it for publication.

## Conflict of Interest

The authors declare that the research was conducted in the absence of any commercial or financial relationships that could be construed as a potential conflict of interest.

## Publisher’s Note

All claims expressed in this article are solely those of the authors and do not necessarily represent those of their affiliated organizations, or those of the publisher, the editors and the reviewers. Any product that may be evaluated in this article, or claim that may be made by its manufacturer, is not guaranteed or endorsed by the publisher.
